# Management of Retained Secundines in Abortion

**Published:** 1879-11-20

**Authors:** 


### *MANAGEMENT OF RETAINED SECUNDINES IN ABORTION.*

Dr. S. Baruch, of Camden, South Carolina, has a paper on this subject in the "Transactions of the South Carolina Medical Association, 1879," which contains some practical suggestions. He finds that only Dr. Fordyce Barker is in entire accord with his views of treatment of such cases. As to the diagnosis of abortion during the first half of pregnancy, experience has taught him one reliable sign, which indicates that the ovum has been dischargad, viz., the funnel-shaped cervix uteri. "By firm pressure, the point of the examining finger can be forced to enter the dilated cervix, and can be made to touch the spasmodically contracted internal os, which presents a sharply defined, rigid, rounded aperture. In the absence of placental remains, this is the only positive evidence of an abortion; it is a condition which neither an ordinary menorrhagia nor any other trouble but an abortion can produce."

Dr. Baruch is satisfied that all attempts to secure removal of the secundines immediately after abortion are harmful and should be abandoned. "The celebrated Dr. Barnes says in one of his excellent lectures, that the reputation of skill in these cases attributed to him is not deserved, because he is usually called in consultation thirty-six to forty-eight hours after the abortion, when the medical attendant has given up in despair his repeated efforts at removal of the secundines. After the expiration of this period, the connection between the foreign body and the uterine tissue has been either greatly softened or entirely severed, and the removal then is a matter of comparative ease."

If hemorrhage threatens, or if the patient cannot be under constant supervision, the tampon should be resorted to. Whether the os is contracted or presents the funnel-shape above alluded to, seal up the cervix, and render it impervious to air and fluid. Depend upon the vaginal tampon as a support for the cervical plug. Give opiates to allay pain when necessary, and ergot as a hæmostatic.

Usually on the second day after the introduction of the plug, the latter is extruded during an act of micturition or defecation, and the retained secundines are expelled with it. Occasionally the latter may hang from the os, whence they can be readily twisted off with forceps. Should the fortunate issue not follow, a vaginal injection of carbolized water is given, and another plug is introduced.—*Obstetric Gazette.*

